# Dissolution of Different Animal Hair Yarn in 1‑Ethyl-3-methylimidazolium Acetate

**DOI:** 10.1021/acsomega.5c03000

**Published:** 2025-05-21

**Authors:** Amjad S. Alghamdi, Peter J. Hine, Michael E. Ries

**Affiliations:** 1 Soft Matter Physics Research Group, School of Physics and Astronomy, 4468 University of Leeds, Leeds LS2 9JT, U.K.; 2 Department of Physics and Astronomy, College of Science, King Saud University, P.O. Box 2455, Riyadh 11451, Saudi Arabia

## Abstract

The partial dissolution of cashmere and merino wool yarns in the ionic liquid 1-ethyl-3-methylimidazolium acetate was studied both with and without pretreatment of the yarns using sodium bisulfite. The cross sections of both yarn fibers were analyzed using optical microscopy for different dissolution times and temperatures. It was found that the dissolution of cashmere yarn (CY) and merino wool yarn (WY) has two competing processes: one rate limited by disulfide bonds and the other rate limited by hydrogen bonds. The yarn dissolution obeyed the time–temperature superposition. From this, two activation energies for each yarn were obtained, one with respect to low temperature (LT) and one for high temperature (HT), *E*_CY LT_ = 110 ± 12 kJ/mol, *E*_CY HT_ = 61 ± 6 kJ/mol, *E*_WY LT_ = 124 ± 14 kJ/mol, and *E*_WY HT_ = 35 ± 1 kJ/mol. The crossover temperature between the low- and high-temperature regimes was found to be 70 °C. The reducing agent (sodium bisulfite) was used to cleave the disulfide bonds in CY and WY. FTIR spectroscopy provided evidence that the disulfide bonds were, in fact, cleaved during this pretreatment. A single linear regime (instead of two) was found on the Arrhenius graphs of the pretreated cashmere (PCY) and the pretreated merino wool yarn (PWY), strongly confirming our hypothesis that at low temperatures, the disulfide bonds determined the rate of dissolution. The subsequent dissolution activation energies were found to be reduced from the low-temperature activation energies for the CY and WY, with their values being *E*_PCY_ = 62 ± 4 kJ/mol and *E*_PWY_ = 66 ± 3 kJ/mol, respectively. With further analysis, the self-diffusion coefficients of [C2mim]­[OAc] for the CY, PWY, and PCY dissolution systems were quantified and compared to the self-diffusion coefficient of pure [C2mim]­[OAc] measured using NMR.

## Graphical abstract

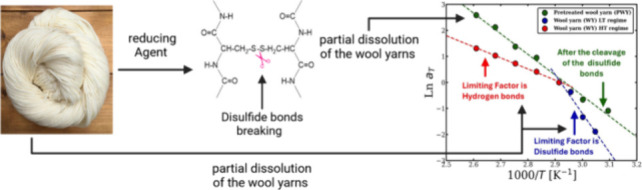

## Introduction

Due to concerns about environmental pollution and the limitations of nonrenewable fossil resources, there is a global pressure to replace petrochemical polymers by more sustainable materials regenerated from biomass resources such as wool, feather, and cellulose.^1,2^ These bioderived materials are environmentally friendly, abundant, and biocompatible.^3^ They also have excellent properties including thermal resistance and mechanical strength.^4^ Annually, more than 1.7 million tons of wool keratin is used in the textile industry worldwide.^5^ Wool keratin is a fibrous cross-linked protein where up to 95% of its weight is pure keratin.^6,7^ It can be viewed as a crystalline intermediate filament reinforced in an amorphous protein matrix, which contributes to its distinctive chemical and physical properties.^8^ It is also distinguished by having a high amount of cysteine, which contains a thiol group (−SH) on its side chain that helps stabilize the three-dimensional structure of proteins.^9,10^ This leads to the formation of a strong covalent disulfide bond that cross-links the polypeptide chains and the matrix molecules with each other and makes them insoluble in water and resistant to some chemical agents.^11^

The dissolution of waste keratin is a primary step to produce regenerated keratin material that can be used in different applications such as bioplastic films,^12^ textile fibers,^13^ and tissue engineering and biomedical materials.^14^ Ionic liquids (ILs) have demonstrated the ability to dissolve different biological macromolecules such as cellulose, silk, and feather and wool keratin.^15–18^ Additionally, they are found to have excellent capability and high efficiency for protein dissolution and recovery.^17–22^ ILs were first reported by Paul Walden in 1914, who investigated the physical properties of ethylammonium nitrates.^23^ ILs can be described as salts consisting of a large organic cation paired with either an organic or inorganic anion, with lower melting temperature than conventional inorganic salts.^24–26^ They have gained significant attention due to their attractive properties, such as low vapor pressure, excellent thermal stability, and nonflammability and can be designed by combining different cation–anion pairs to create the optimal IL for the intended application.^27^ In 2005, Xie et al. first used ILs to dissolve wool keratin.^28^ Recently, the capabilities of many ILs in dissolving keratin were examined using a conductor-like screening model for realistic solvation (COSMO-RS) simulation, revealing that 1-ethyl-3-methylimidazolium acetate dissolved 38 wt % of wool at 120 °C and 1-butyl-3-methylimidazolium chloride dissolved 35 wt % of wool at a higher temperature of 180 °C.^24^ To initiate the dissolution of keratin-based materials, 65% of the disulfide bonds should be broken.^29^ In terms of IL recyclability, [C_4_C_1_im]­[C_1_CO_2_] was successfully recycled and reused three times, without major impact on the dissolution and recovery process.^17^

Several studies have been conducted on the dissolution kinetic of cellulose-, silk-, and wool-based yarns using time–temperature superposition.^30–37^ Cellulose and silk dissolution followed an Arrhenius behavior with one activation energy. However, in our previously published work, we found that the dissolution of wool keratin had two activation energies. The Arrhenius plot was fitted with two linear relations with a crossover temperature at 70 °C.^37^ The lower-temperature regime is rate limited by disulfide bonds, while the higher-temperature regime above 70 °C is rate limited by hydrogen bonds. This is due to the high content of cross-linked disulfide bonds in keratin-based materials, especially in the scale layers (the outer layer of the wool fiber).^37,38^ Treatments such as sodium bisulfite [NaHSO_3_] can be used to disrupt the disulfide bonds on the surface of wool keratin.^39–42^

In the present work, we investigated the dissolution of cashmere and merino wool both as received and when pretreated with NaHSO_3_, by the IL [C2mim]­[OAc]. The effect of the pretreatment was compared to our previously published work on the same untreated fiber.^37^ Optical microscopy (OM) was used to track the decrease in the cross-sectional area of the dissolved yarns as a function of the temperature and time. Then, time temperature superposition (TTS) analysis was employed to calculate the activation energy of the dissolution of the yarns. The reduction in the yarn area was used to determine the thickness loss of the dissolved yarn with time and temperature. This allowed the self-diffusion coefficient of [C2mim]­[OAc] in the dissolving yarn to be determined. This study provides insight into the dissolution of keratin in ILs, leading to an enhanced understanding of how to recycle animal hair waste from textiles and optimize the dissolution process for the formation of biocomposites.

## Materials and Methods

### Materials

Natural undyed Merino wool with an approximate filament diameter of 30 ± 5 μm was purchased from the ″80 Skeins” online yarn shop in Rugby, United Kingdom. Undyed natural white 100% Mongolian cashmere was supplied by “Brian’s Best Wools”, Keighley, West Yorkshire, United Kingdom, with a filament diameter on the order of 18 ± 2 μm. These two types of wool were used as a source of keratin protein and kept at room temperature in a dry place.

1-Ethyl-3-methylimidazolium acetate ([C2mim]­[OAc], purity ≥98%) was purchased from Proionic GmbH, Grambach, Austria. The water content of [C2mim]­[OAc] was measured to be <0.2% using a Karl Fischer titration apparatus (899 Coulometer, Metrohm U.K. Ltd., UK). The reducing agent sodium bisulfite [NaHSO_3_] was purchased from Sigma-Aldrich, Gillingham, United Kingdom. To have a clear optical image for the cross-sectional yarn area, a cold-curing epoxy resin bought from “EpociCure 2” Buehler, Coventry, UK, was used.

### Methods

#### Pretreating and Dissolving Merino Wool and Cashmere Yarns

First, cashmere yarn was cut into eight threads, each 15 cm long, and then these threads were wound around a picture frame with a size of 8 cm × 8 cm square made of Teflon. Then, the frame/yarn was put into a Teflon tray with excess [C2mim]­[OAc], which was heated beforehand for 1 h (the mass ratio of yarn to IL was 1:40). After that, the tray was placed into a preheated vacuum oven for an hour (Sheldon 17L Digital Vacuum Oven SQ-15VAC-16, Sheldon Manufacturing, Inc., USA). The dissolution process took place under vacuum to prevent the moisture uptake of [C2mim]­[OAc], which affects its properties and dissolution process.^43–46^ After the frame/yarn was removed from the oven, it was soaked in a water bath to coagulate the dissolved keratin and to wash any remaining IL from the sample. Finally, the yarn samples were dried for 2 days at room temperature and then cut free from the frame and prepared using an epoxy resin for the optical measurement (see Figure 1a).

**Figure 1:**
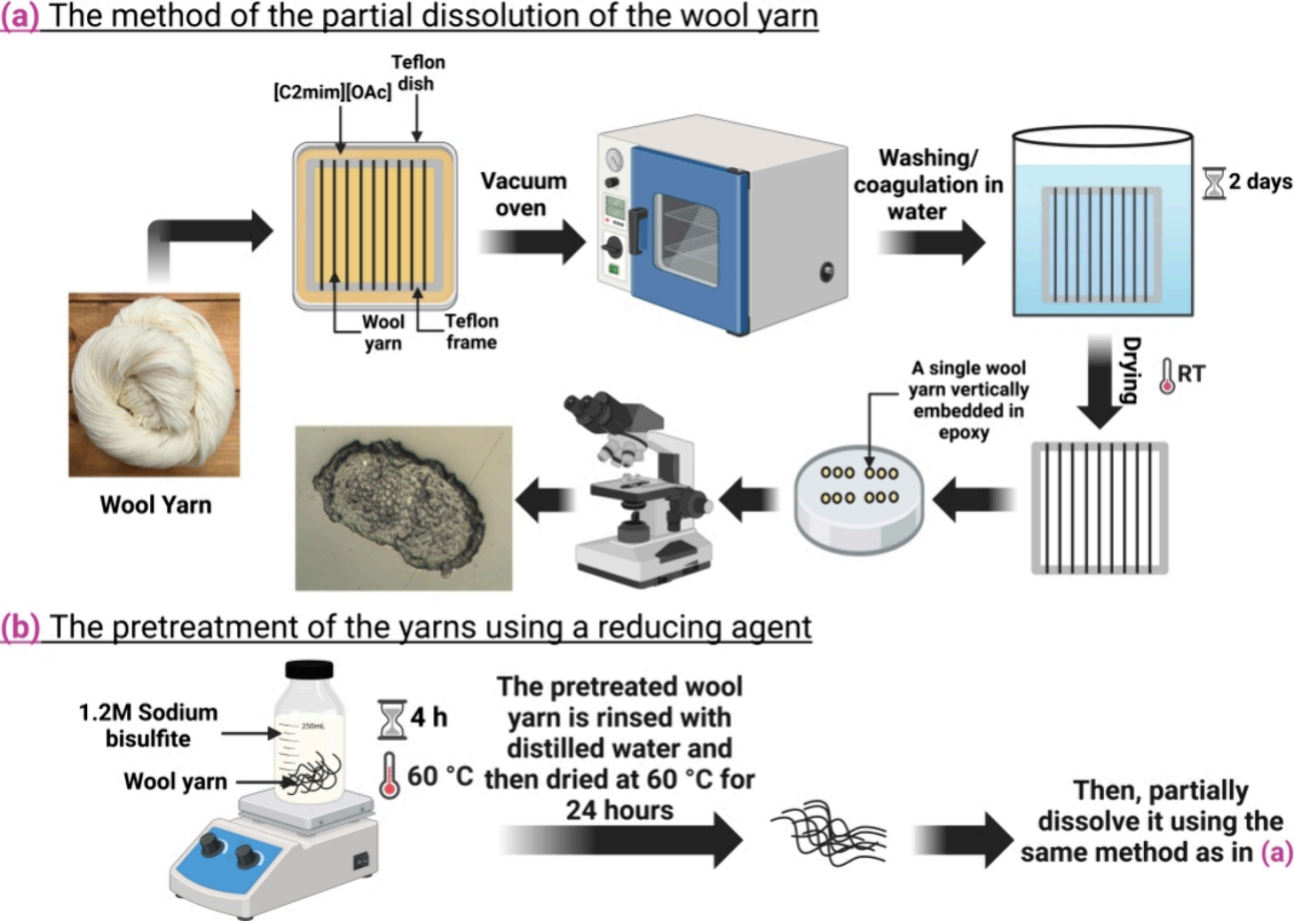
Schematic showing (a) the preparation process from the partial dissolution process to the optical characterization of merino wool/cashmere yarns and (b) the pretreatment method using NaHSO_3_ as a reducing agent. Created in BioRender (https://BioRender.com/m09z243).

In the second part of the experiment, Merino wool and cashmere yarns were subjected to a pretreatment with a reducing agent NaHSO_3_ to cleave the disulfide bonds in the keratin. Both yarns were soaked separately in 1.2 M NaHSO_3_ (with a solid-to-liquid ratio of 1:125) at 60 °C for 4 h and then rinsed with distilled water for 24 h and dried for a day at 60 °C.^39^ The dried pretreated Merino wool and cashmere yarns were prepared in the same way as the previous cashmere yarn samples for dissolution using [C2mim]­[OAc] solvent (see Figure 1b).

#### FTIR Characterization of Merino Wool and Cashmere

The difference between Merino wool and cashmere yarns before and after the pretreatment of NaHSO_3_ was investigated using PerkinElmer Spectrum One Fourier transform infrared spectroscopy (FTIR) (PerkinElmer, UK). For each spectrum, 100 scans were recorded in the spectral region between 4000 and 550 cm^–1^ with a resolution of 4 cm^–1^ and a scan speed of 0.5 cm/s.

#### Optical Microscopy

For optical examination, the yarn samples, both raw and processed at different temperatures and times, were fixed vertically in a silicon mold. Then, they were encapsulated in epoxy resin (4:1 of epoxy resin and hardener) and allowed to cure for 24 h. To have a clear cross-sectional image of the yarn, the surface of the prepared epoxy resin was ground and polished, finishing with 1 μm alumina paste.

Optical microscopy is used as a technique to investigate the dissolution process of the keratin yarn. An Olympus BH2 microscope (Olympus Corporation, Tokyo, Japan) was utilized in reflection mode coupled with a charge-coupled-device camera to capture the cross-sectional of yarn. To accurately measure the cross-sectional area of different yarns, ImageJ software (version 1.53s) was used.

#### Calculating the Keratin Thickness Loss and the Diffusion Coefficient of the Ionic Liquid through the Partially Dissolved Yarn

During the dissolution of the wool and cashmere yarns, it was noticeable that some of the keratin material was lost in the excess [C2mim]­[OAc] (see Figure 2). The thickness loss of dissolved keratin can be expressed as a function of time and was mathematically modeled by measuring the effective mean square radius for the irregularly shaped yarn. Thus, to calculate the thickness loss as a function of time (*x*_rms_(*t*)), we used the following equation:

$$x_{\mathrm{rms}}(t)=(A(0)/\pi {)}^{1/2}-(A(t)/\pi {)}^{1/2}$$ 1

where *A*(0) and *A*(*t*) are the cross-sectional area of yarn before the dissolution and after the dissolution, respectively, at different dissolution temperatures and times. If the linear relationship between the modeled thickness loss *x*_rms_ and the square root of the dissolution time holds, then the *x*_rms_ can be modeled by the mean square displacement of a particle in one dimension. So, the self-diffusion coefficient *D* of [C2mim]­[OAc] through the wool and cashmere yarn at different times *t* can be calculated using

$$x_{\mathrm{rms}}^{2}=2Dt$$ 2

$$x_{\mathrm{rms}}=(2D{)}^{1/2}t^{1/2}$$ 3

**Figure 2:**
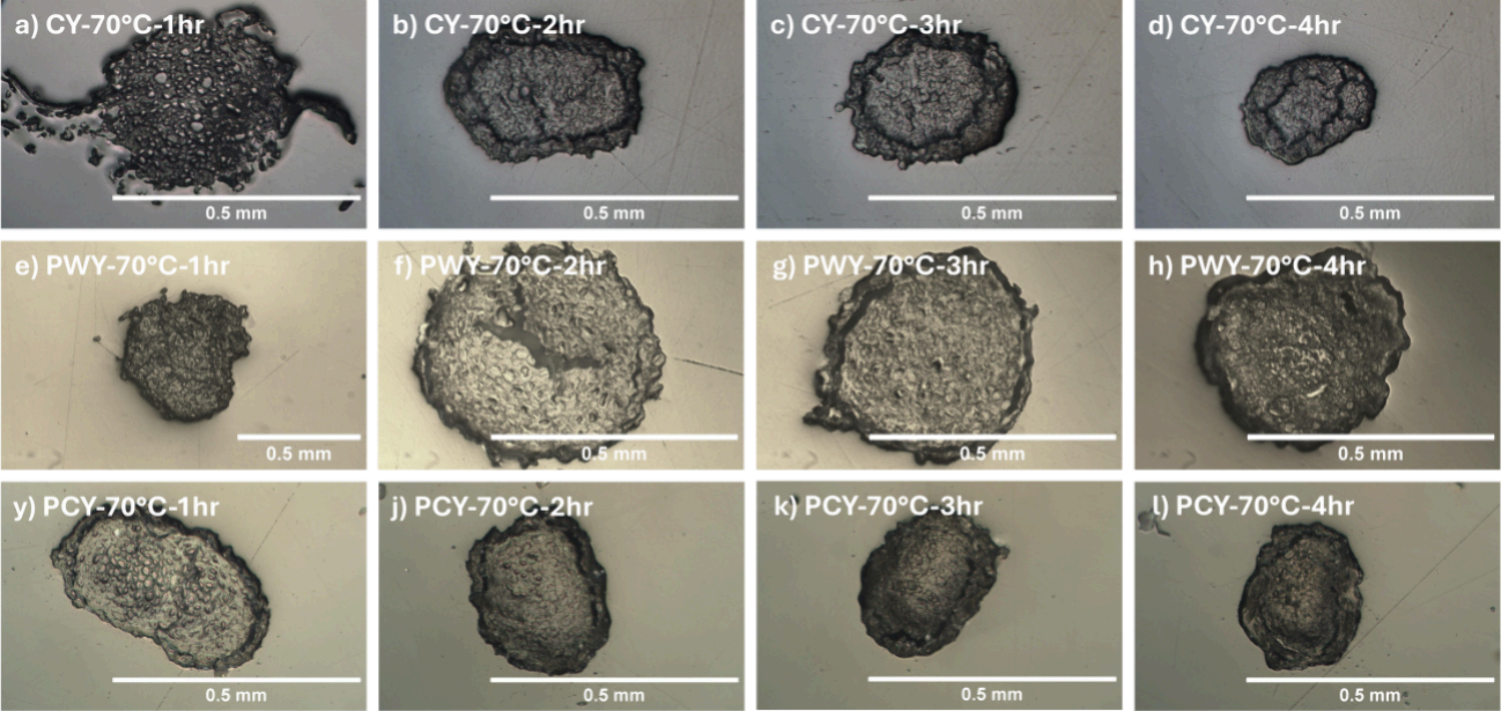
Optical images of partially dissolved yarn at 70 °C for different times from left to right 1, 2, 3, and 4 h. (a–d) Cashmere yarn (CY), (e–h) pretreated wool yarn (PWY), and (i–l) pretreated cashmere yarn (PCY). These images show how the cross-sectional area reduces in size as the processing time increases.

## Results and Discussion

### Optical Microscopy

The cashmere yarn (CY), pretreated cashmere yarn (PCY), and pretreated wool yarn (PWY) consist of individual nonbonded multifilaments. After dissolution in [C2mim]­[OAc] and then coagulation in the water, it can be seen from Figure 2 (for example, at a temperature of 70 °C) that the yarn multifilaments become tightly packed. This is because some of the dissolved then coagulated keratin glues the multifilaments together, with some of the dissolved keratin being dissolved into [C2mim]­[OAc]. As the keratin protein consists of different amino acids, some of these groups are hydrophilic and some are hydrophobic; as a result, when wool keratin is subjected to dissolution, this could introduce material soluble in water.^18^ It was noticeable from the optical images that as the processing temperature increased and the dissolution time progressed, the cross-sectional area of the yarn became smaller. Therefore, the parameter used to track the yarn dissolution and calculate the yarn dissolution activation energy was the remaining cross-sectional area. By using the ImageJ software, the cross-sectional area was measured by drawing a line around each individual filament in the yarn, including the nonbonded filaments as seen at some samples processed at low temperature or short time (see Figure 2a). Keratin yarn does not have a coagulated ring around the undissolved material core as reported for cellulose-based yarns such as flax, cotton, and hemp.^30,35,47^ It was reported in a previous study on Merino wool that SEM images of the partially dissolved yarn at 70 °C for different time periods showed that the structure of individual keratin fibers in the outer layer of the yarn remained the same as that of the undissolved yarn filaments.^37^

### Time–Temperature Superposition of Cashmere Yarn

First, the dissolution of as-received (and untreated) cashmere yarn (CY) was studied at different temperatures and dissolution time periods. At each processing temperature and time, six yarn optical images were recorded to measure the cross-sectional area change. Figure 3a illustrates how the overall cross-sectional area of CY decreased with the temperature and time. To study the dissolution behavior of CY, a range of temperatures were investigated from what we term the low-temperature regime (LT), including 50, 55, 60, 65, and 70 °C, and the high-temperature regime (HT), including 70, 80, 90, 100, and 110 °C. This temperature range was chosen to compare the dissolution behavior of CY with our previously published study done on the dissolution behavior of untreated Merino wool.^37^ In the previous study, we found that by tracking the reduction of the cross-sectional area of the Merino wool yarn and employing time–temperature superposition (TTS), the dissolution activation energy could be calculated. This study showed that the system had two activation energies: one for the low-temperature regime and one for the high-temperature regime. Time–temperature superposition is an analytical method often used in rheology studies. It has previously been used in our research group for tracking the dissolution of different types of cellulose-based yarns, regenerated cellulose, silk, and keratin yarns.^30,33,36,37,48,49^

**Figure 3:**
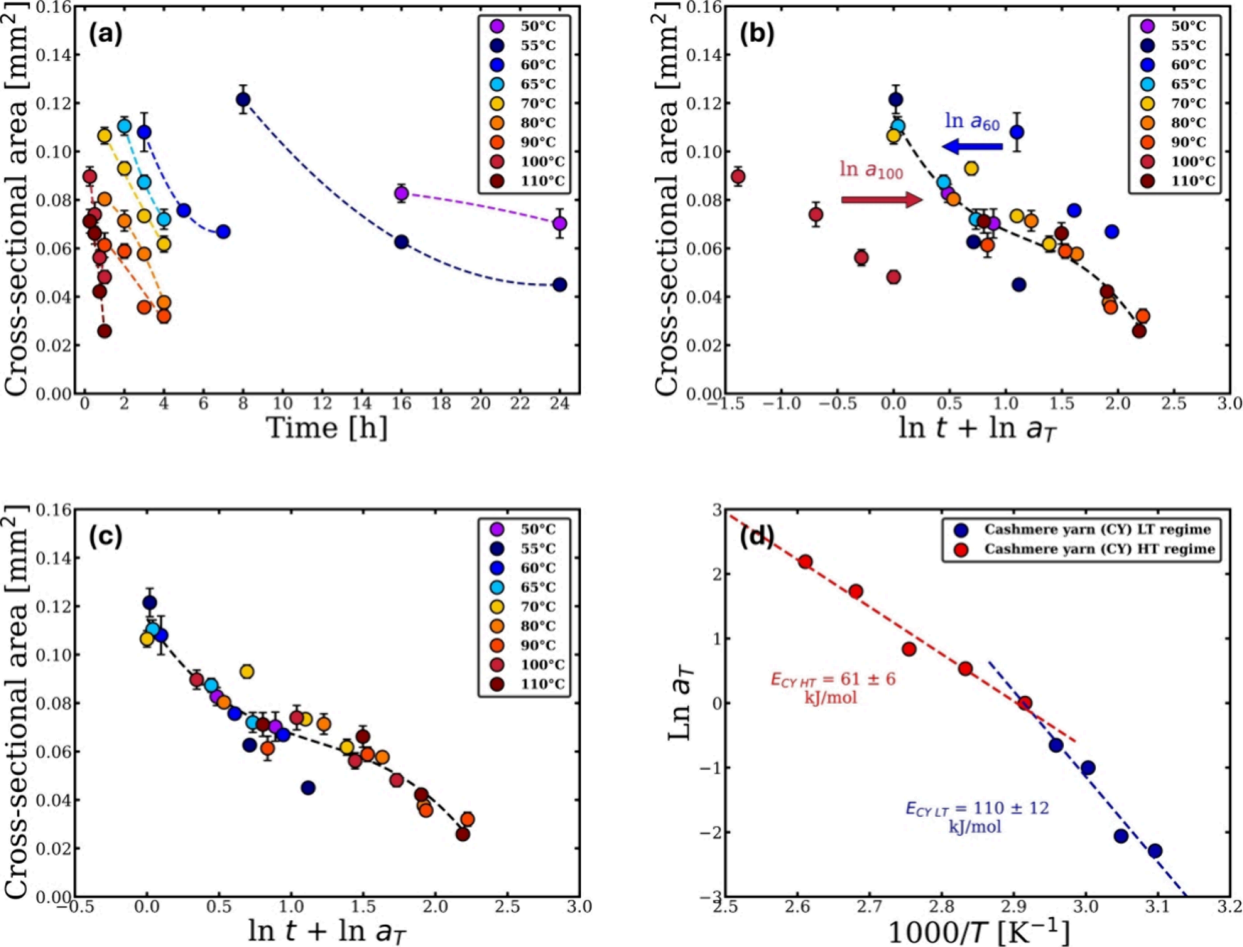
(a) Change in the cross-sectional area of CY with temperature and time. (b) How to shift each set of data at each temperature horizontally to overlap with the data at 70 °C. (c) Time–temperature superposition plot at the reference temperature 70 °C. (d) Shift factor ln *a*_T_ vs the inverse temperature, where in this case, the data fits with two linear equations indicating two Arrhenius behavior with a cross over temperature at 70 °C. All the errors were calculated, but in some cases, these are smaller than the point size.

Here, we analyzed the data of CY using TTS by creating a master curve, which involved several steps. The *x*-axis scale was converted from a linear time scale (*t*) into a logarithmic scale (ln *t*) for all data sets. Figure 3b illustrates the construction of the master curve, where the data set at 70 °C (yellow data points) was chosen as the reference temperature. Since different temperatures affect the dissolution rate differently, each data set must be shifted horizontally along the logarithmic time axis to overlap with the reference data set at 70 °C using eq 5.

$$t_{T}^{\prime }=t_{T}a_{T}$$ 4

$$\ln\;t_{T}^{\prime }=\ln\;t_{T}+\ln\;a_{T}$$ 5

where *t*_T_^′^ is the shifted time, *t*_T_ is the original time, and *a*_T_ is the scaling factor. The shift for each temperature is obtained by adjusting each data set horizontally until they overlap with the reference data set (at 70 °C) as closely as possible This shift is determined using a shift factor (ln *a*_T_), which takes into consideration the variations in dissolution behavior at different temperatures, as schematically shown in Figure 3b for the data sets at 60 and 100 °C. To ensure optimal overlap, the combined data sets were fitted with a cubic function, and the regression coefficient *R* ^2^ was maximized by computing the shift factor using Excel.

Afterward, the rate of the dissolution can be examined by plotting the shifting factor ln *a*_T_ obtained for each temperature in the system against the reciprocal of the temperature in kelvin. The Arrhenius graph in Figure 3d shows two regimes: one dominates at temperatures below 70 °C (low-temperature (LT) regime), and another dominates at temperatures above 70 °C (high-temperature (HT) regime). Two dissolution activation energies were calculated for the untreated cashmere yarn (CY) in [C2mim]­[OAc], *E*_CY LT_ = 110 ± 12 kJ/mol and *E*_CY HT_ = 61 ± 6 kJ/mol, for the low-temperature regime and the high-temperature regime, respectively, using the following Arrhenius equations.^50^

$$a_{T}=A\exp(-E_{a}/RT)$$ 6

$$\ln\;a_{T}=\ln\;A-E_{a}/RT$$ 7

where *E*_a_ is the Arrhenius activation energy, *A* is the Arrhenius pre-exponential factor, *R* is the gas constant, and *T* is the temperature in kelvin. The two regimes were also found in our previously published study on the dissolution of Merino wool by using exactly the same experimental method and analysis. Interestingly, the Merino wool system had a sharper transition between the two temperature regimes at 70 °C with a lower activation energy for the high-temperature regime of 34 ± 1 kJ/mol but a similar activation energy for the low-temperature regime of 127 ± 9 kJ/mol.^37^ We suggest that the dissolution rate is controlled by the slowest factor at any temperature, which determines the activation energy. Our hypothesis suggests that at a lower temperature, the disulfide bonds, which have a higher activation energy, are the limiting factor. Conversely, at a higher temperature, the hydrogen bonds, which have lower activation energy, become the limiting factor. So, here in the CY/[C2mim]­[OAc] dissolution system, 70 °C is believed to be the crossover temperature where the two regimes equally contribute to the reaction. To ensure that 70 °C was the crossover between the regimes, TTS master curves were generated at different reference temperatures, 60, 65, 80, and 90 °C. Four different TTS master curves were obtained, and each corresponding Arrhenius data were fitted with two linear equations, where the crossover temperatures were 60, 65, 80, and 90 °C. The crossover temperature was determined by calculating the regression coefficients *R* ^2^ for each Arrhenius line (LT and HT regimes) separately. The average value of these two *R* ^2^ is found to be at its maximum value when the crossover temperature is set to 70 °C, as seen in Table 1. Table 1 includes all of the dissolution activation energies for CY [C2mim]­[OAc] and the average (*R* ^2^) values at different crossover temperatures.

**Table 1:** Two-Regime Arrhenius Plot Fitting Result for CY Dissolution

crossover temperature (°C)	*E*_CY LT_ (kJ/mol)	*E*_CY HT_ (kJ/mol)	regression coefficient *R* ^2^
60	114 ± 44	66 ± 4	0.9282
65	111 ± 20	65 ± 5	0.9601
70	110 ± 12	61 ± 6	0.9697
80	96 ± 10	66 ± 9	0.9626
90	81 ± 9	78 ± 13	0.9565

### Separate TTS Analysis for the Low-Temperature Regime and the High-Temperature Regime

To further examine these two regimes, we analyzed the low-temperature (≤70 °C) and high-temperature (≥70 °C) regimes independently, instead of using a single reference temperature for both regimes of 70 °C. First, using the same analysis method introduced above, the low-temperature regime was studied (50–70 °C) with chosen 60 °C in the middle of the LT regime temperature range as the reference temperature. Then, the data in Figure 4b were fitted with a polynomial function and other data sets at each temperature were shifted by using a scaling factor (*a*_T_) to overlap with the data point at 60 °C. Additionally, the best overlapping of all data sets to 60 °C was achieved by solving until the value of the regression coefficient reached its maximum. The master curve can be plotted in real time by calculating the shifted time using eq 4 (Figure 4c).

**Figure 4:**
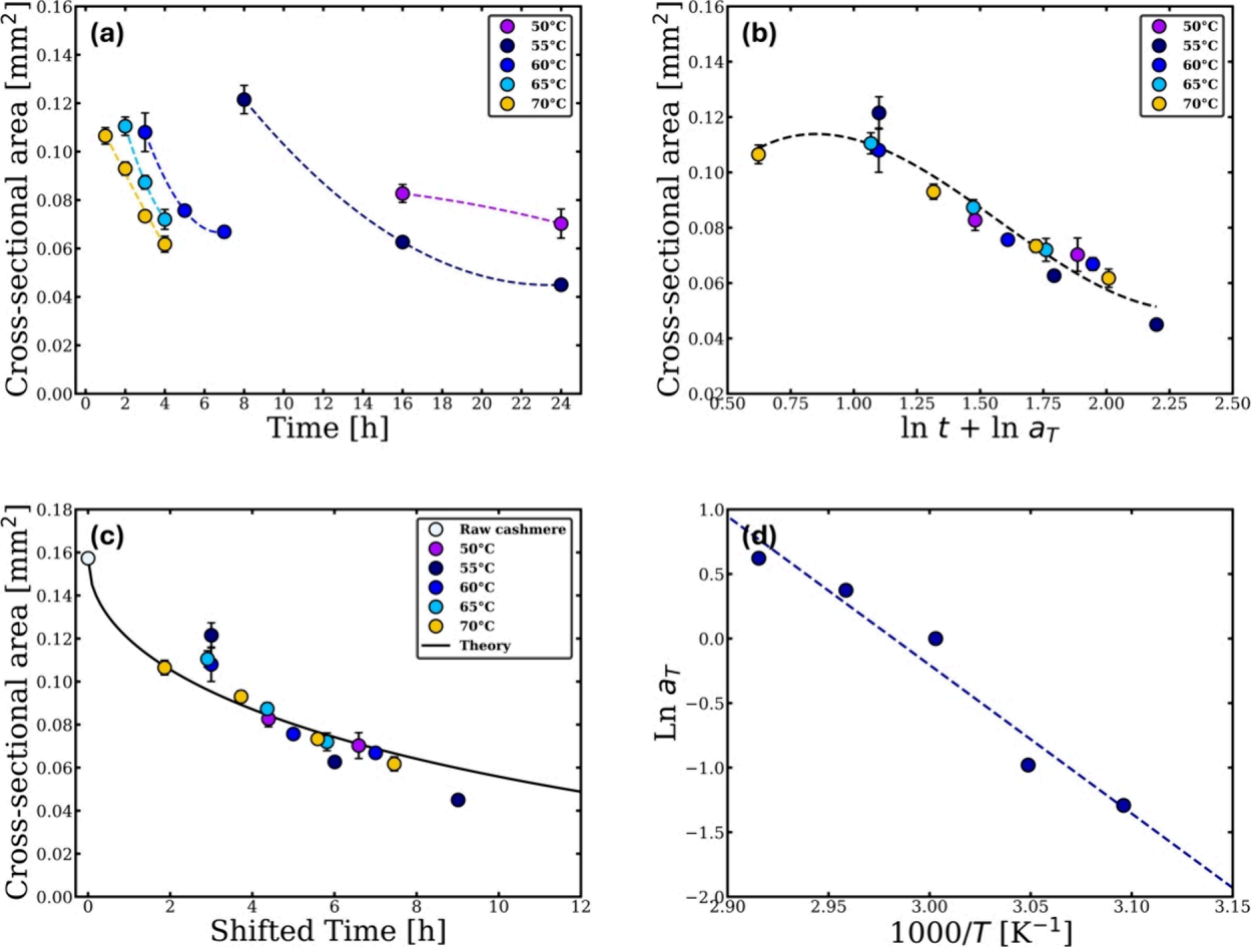
(a) Cross-sectional area changes with temperature and time. (b) Time–temperature superposition plot at the reference temperature 60 °C. (c) Real dissolution time TTS curve at 60 °C. (d) Arrhenius graph showing one linear relation. All the errors were calculated, but, in some cases, these are smaller than the point size.

The shifting factor plotted versus the reciprocal of the temperature, which shows an Arrhenius-like behavior with a single activation energy calculated using eq 7, is 96 ± 12 kJ/mol. This analysis gave a similar value of the dissolution activation energy in the LT regime as that derived from the unified master curve data. This consistency confirms that the activation energy is the same in both analysis methods and demonstrates the methodology robustness.

Next, the high-temperature (HT) regime, which includes the temperatures 70, 80, 90, 100, and 110 °C, was analyzed using the same method. Figure 5 shows the same steps again to eventually calculate the dissolution activation energy for the HT regime of CY. The master curve is constructed at the reference temperature of 90 °C. The dissolution activation energy was found to be 67 ± 4 kJ/mol, which is close to that obtained using the two-regime analysis (Figure 3d).

**Figure 5:**
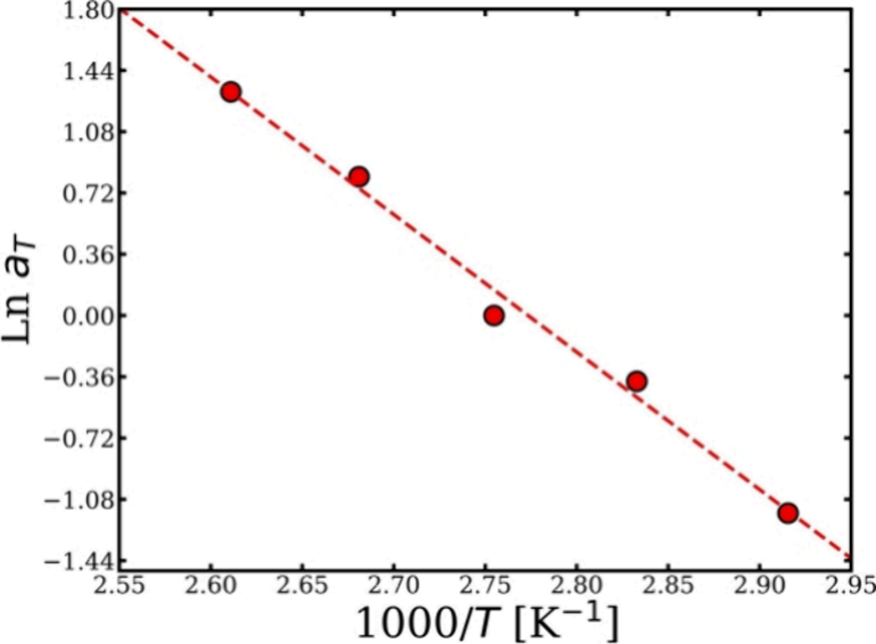
Arrhenius plot showing the relation between the ln *a*_T_ and temperature, with the data fitted to a linear relation using 90 °C as a reference temperature. All the errors were calculated, but, in some cases, these are smaller than the point size.

### Modeling Yarn’s Thickness Loss

An interesting further analysis can be done using the data in Figure 4c, and a similar figure can be produced from the HT regime data, which represents the real-time master curve of the reduction in the cross-sectional area of the cashmere yarn dissolved in [C2mim]­[OAc] using different reference temperatures 60 and 90 °C, respectively. It can be noticed from Figure 4c that the rate of material loss slows with time. Figure 6 illustrates the relation between *x*_rms_ with the square root of the shifted time at two different reference temperatures 60 and 90 °C, which represent the low- and high-temperature regimes, respectively.

**Figure 6:**
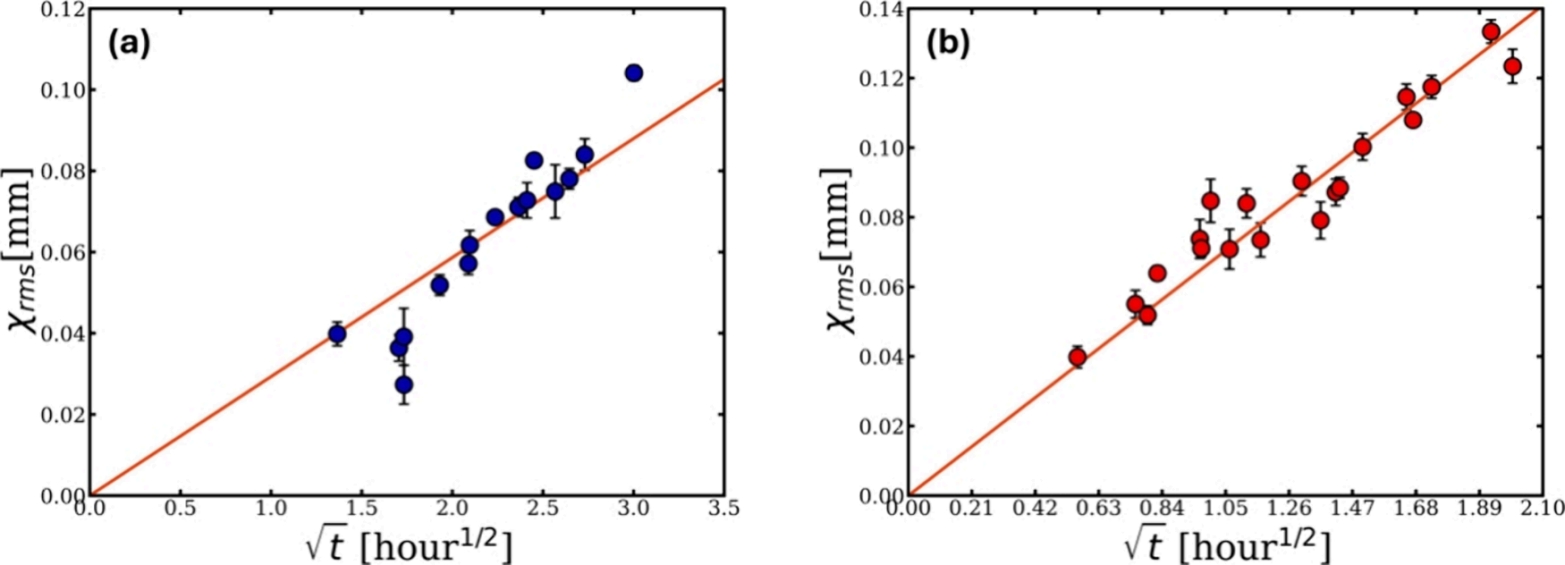
Plot of the thickness loss *x*_rms_ of the CY at (a) 60 °C and (b) 90 °C as a function of $\sqrt{t}$ .

These two plots were obtained using the linear time scale master curves at 60 °C (shown in Figure 4c) and 90 °C. In the CY dissolution system, a total of nine master curves should be produced, each corresponding to a reference temperature within the system, representing both the LT and the HT regimes. Afterword, thickness loss *x*_rms_ can be calculated using eq 1 for each master curve and then plotted against the square root of real time.

From Figure 6, the values of *D* can be calculated where a linear fit can be applied to this relationship, with the gradient used to calculate the self-diffusion coefficient *D* according to the diffusion equation for the Brownian motion (eq 3). This implies that the thickness loss is controlled by diffusion and the *D* value at each temperature are listed in Table 2.

**Table 2:** Self-Diffusion Coefficients *D*_[C2mim][OAc]_ for the Cashmere Yarn at Different Reference Temperatures

reference temperature (°C)	*D*_[C2mim][OAc]_ (10^–13^ m^2^ s^–1^)
50	0.33 ± 0.01
55	0.45 ± 0.02
60	1.19 ± 0.04
65	1.74 ± 0.06
70	2.71 ± 0.06
80	4.25 ± 0.08
90	6.3 ± 0.1
100	14.1 ± 0.3
110	23.3 ± 0.4

The values of the *D*_[C2mim][OAc]_ summarized in Table 2 are quite consistent with the values obtained in previous studies using proton nuclear magnetic resonance (^1^H NMR), where the *D* of the cation and the anion in the pure IL are measured separately, with *D*_[C2mim]_ = 9.6 ± 0.2 × 10^–12^ m^2^/s and *D*_[OAc]_ = 7.7 ± 0.4 × 10^–12^ m^2^/s, respectively.^51^ The values of *D* in this work are also comparable with the value obtained in the work by Alrefaei et al. where the *D* of [C2mim]­[OAc] ions diffusing at 40 °C through the saturated layer of cellulose was calculated to be 6.74 × 10^–13^ m^2^/s.^35^ As expected, as the dissolution temperature increased, the self-diffusion coefficient *D*_[C2mim][OAc]_ increased, and it is lower than that found in the NMR work on pure [C2mim]­[OAc] due to the presence of the cashmere yarn.

The data in Table 2 can be used to plot ln *D* as a function of 1/T to obtain the diffusion activation energy of the CY. Figure 7 illustrates that two diffusion activation energies can be calculated by fitting a linear relationship with respect to each regime, which gives a similar interpretation to that found when the dissolution activation energies were calculated (see Figure 3d). The diffusion activation energy for each regime was calculated using the following equation.

$$\ln\;D=\ln\;D_{0}-E_{D,a}/RT$$ 8

where *D*_0_ represents the pre-exponential factor and *E*_D,a_ is the activation energy of diffusion. Two diffusion activation energies were obtained for the CY dissolution system: for the low-temperature (LT) regime, it was *E*_D CY LT_ = 96 ± 12 kJ/mol, and for the high-temperature (HT) regime, it was *E*_D CY HT_ = 64 ± 4 kJ/mol. These energies are very close to the dissolution activation energies of CY in [C2mim]­[OAc].

**Figure 7:**
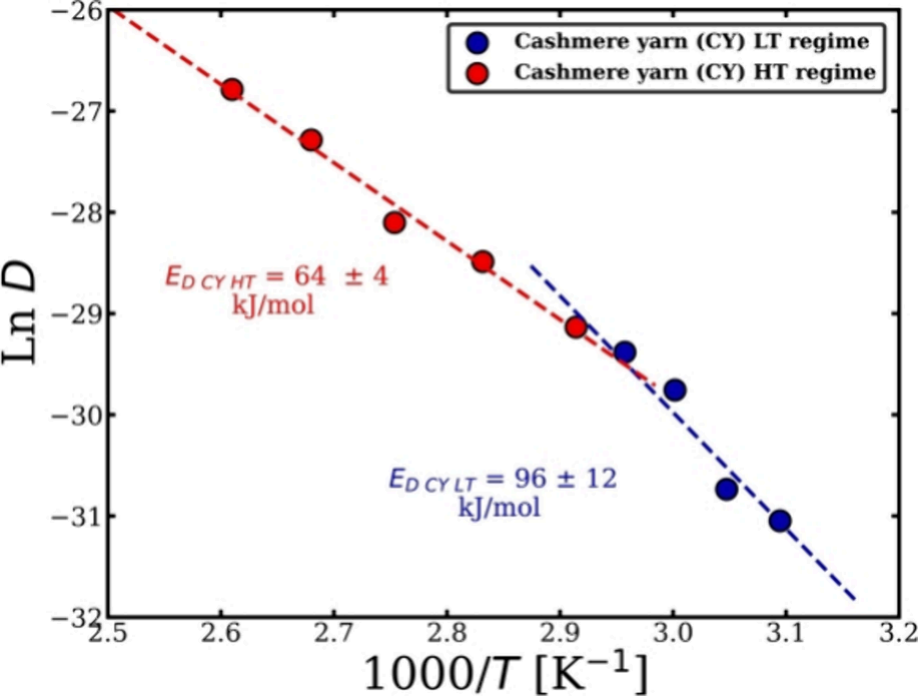
ln *D* versus 1000/T, presenting two Arrhenius behavior each regime the low and high temperatures with a crossover temperature at 70 °C. All the errors were calculated, but in some cases, these are smaller than the point size.

### FTIR-Pretreated Merino Wool and Cashmere Yarn

In our previous published paper, we investigated the dissolution kinetics of untreated Merino wool in [C2mim]­[OAc].^37^ This work showed that the dissolution had both low- and high-temperature regimes, which we hypothesized to be controlled by disulfide bonds and hydrogen bonds, respectively. The current work presented above, investigating cashmere yarn dissolving in [C2mim]­[OAc], confirmed this behavior for a different animal hair. To test our idea that disulfide bonds give rise to the two activation energies, we used NaHSO_3_ as a reducing agent, to cleave the disulfide bonds and remove or reduce its effect in the dissolution process in the low-temperature regime. The method of pretreatment and the preparation of the partial dissolution of yarns were explained above. It was assumed that no weight loss occurred during the pretreatment of wool with NaHSO_3_ because the concentration of the solution was not high. The cleavage of the disulfide bonds was investigated using an FTIR spectrometer, with results shown in Figure 8.

**Figure 8:**
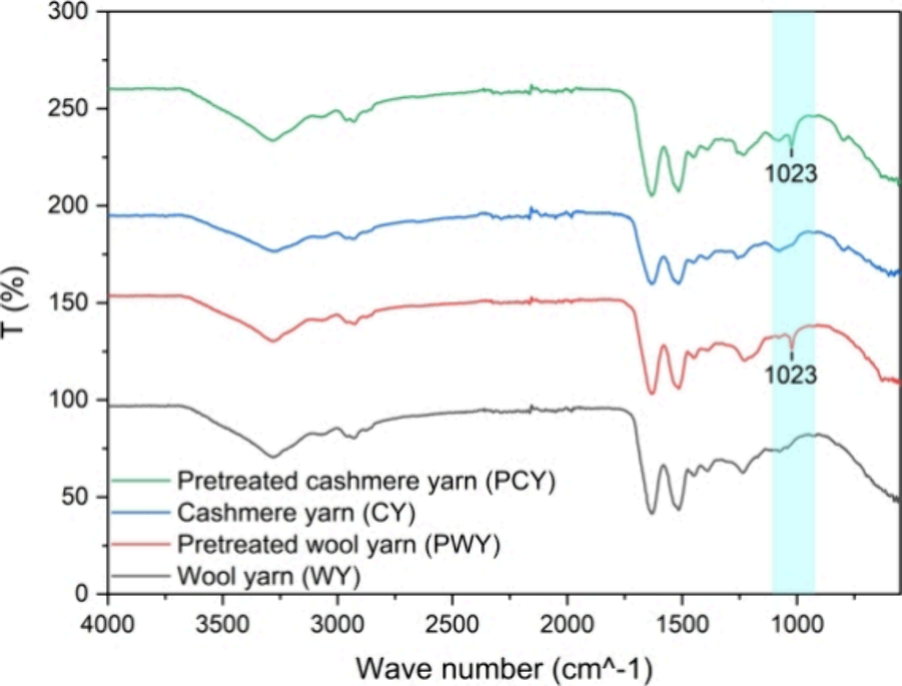
Fourier-transformation (FTIR) spectra of Merino wool yarn (WY) (black), pretreated Merino wool yarn (PWY) (red), cashmere yarn (CY) (blue), and pretreated cashmere yarn (PCY) (green).

The FTIR analysis of the WY and PWY showed a notable distinction in the PWY spectrum, which is a higher intensity at around 1023 cm^–1^. The same observation was detected for the CY and the PCY. This intensity increase is related to the presence of the cysteamine sulfonate (−SSO_3_) groups,^41,52,53^ which suggests that disulfide bonds (−S–S−) in wool have undergone cleavage during the NaHSO_3_ chemical treatment generating (−SH) bonds that were oxidized in the air, without disturbing the main chemical backbone of the keratin protein.^41^

### TTS of Pretreated Wool Yarn

The dissolution of pretreated merino wool yarn (PWY) in [C2mim]­[OAc] was tracked through the decrease in the cross-sectional area at different temperatures and times using the optical images (see Figure 2e–h). The exact time–temperature superposition analysis method explained in detail above to study the dissolution of CY was used here as well. The data of the PWY (in green) on the Arrhenius graph (Figure 9) shows one dissolution regime instead of two regimes for the merino wool yarn, which are plotted on the graph in blue and red (the dissolution of the nonpretreated merino wool yarn is explained in detail in a previous publication).^37^ The dissolution activation energy of the PWY is calculated to be *E*_PWY_ = 66 ± 3 kJ/mol using eq 7). This activation energy is almost half of the calculated activation energy for the low-temperature (LT) regime (rate limited by disulfide bonds) of nonpretreated wool, which is *E*_WY LT_ = 124 ± 14 kJ/mol. Surprisingly, it is found to be higher than the dissolution activation energy of the high-temperature (HT) regime (rate limited by hydrogen bonds), which is *E*_WY HT_ = 35 ± 1 kJ/mol. We suggested that the reaction between a disulfide bond and NaHSO_3_ gives a thiol group, which can hydrogen bond through the (−S–H) group.^54^

**Figure 9:**
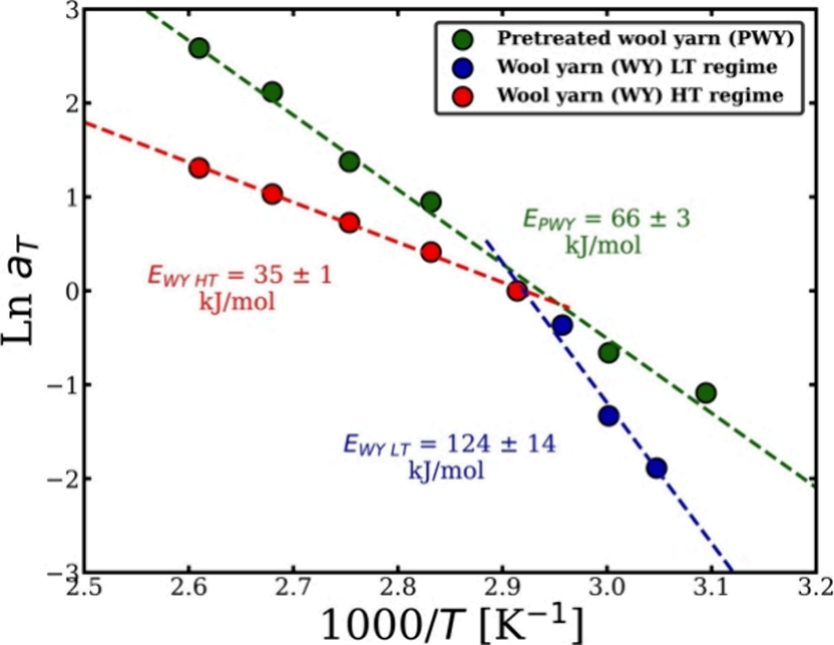
Arrhenius graph at the crossover temperature 70 °C of the merino wool yarn (WY) without the reducing agent (RA) pretreatment with two dissolution regimes, one at low temperature (LT) and the other at high temperature (HT); the green line represents the Arrhenius plot of the dissolution process of pretreated wool yarn (PWY). All the errors were calculated, but, in some cases, these are smaller than the point size.

Figure 10 shows how the dissolution rate of the wool yarn at each temperature used in the dissolution system varies relative to pretreated merino wool yarn. The relative dissolution rate can be calculated using the difference between the shift factor ln *a*_T_ of the WY and the PWY using the data in Figure 9. Interestingly, the dissolution rate of the PWY is significantly faster at each temperature except at 70 °C where the dissolution rate is the same as the wool yarn, where it has been suggested that both proposed processes LT and HT regimes are equally contributed and was chosen to be the crossover temperature between the two regimes.^37^

**Figure 10:**
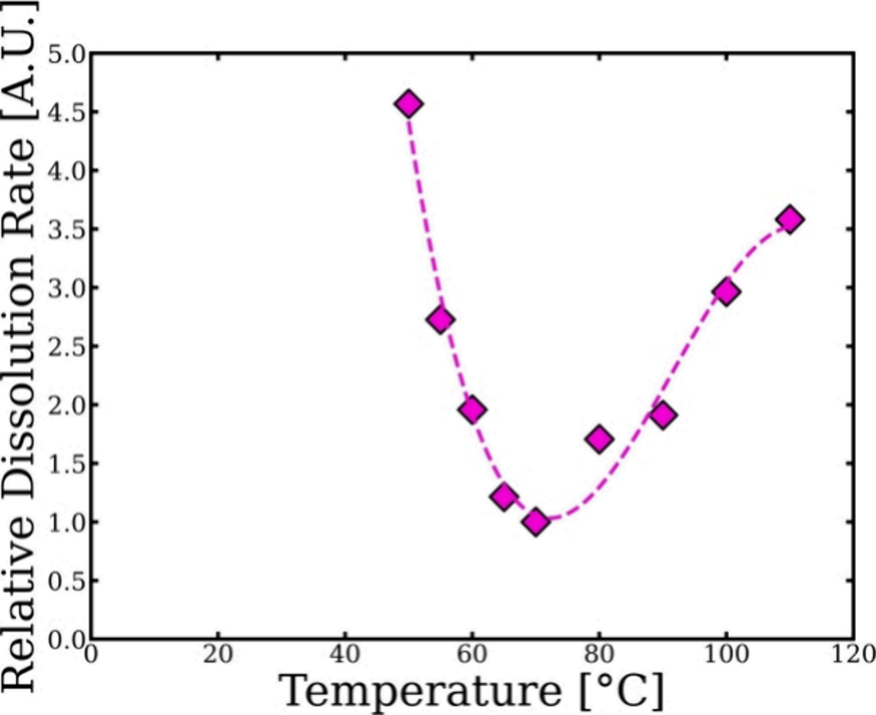
Relative dissolution speed of the pretreated merino wool yarn in relation to the merino wool yarn as a function of temperature. Polynomial fit was used to guide the eye.

The self-diffusion coefficients *D* of [C2mim]­[OAc] through the PWY were calculated at each temperature in the system (50, 60, 70, 80, 90, 100, and 110 °C) using the analysis explained in Modeling Yarn’s Thickness Loss, where the real-time master curve is first obtained by using each temperature within the system as a reference temperature, as an example the real-time master curve at 70 °C. Next, the thickness loss of the yarn was calculated using eq 1. The plot of the (*x*_rms_) and the square root of the real time once again fitted well with a linear relation where *D* can be calculated from the gradient using eq 3 (see Figure 11a and Table 3).

**Figure 11:**
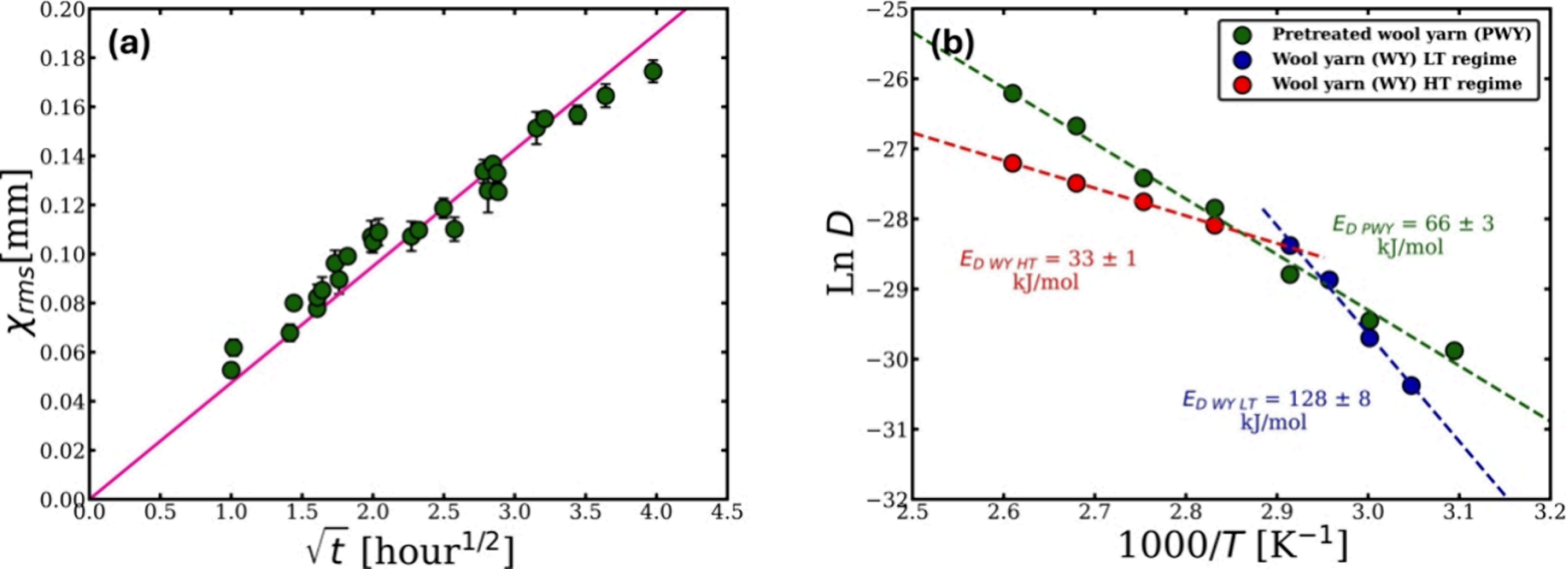
(a) This graph shows the linear relation between the square root of the real-time master curve at 70 °C and the modeled thickness loss of the processed PWY yarn. (b) Arrhenius plot illustrates one regime and two regimes for the diffusion activation energy of the processed PWY and WY in [C2mim]­[OAc], respectively. All the errors were calculated, but in some cases, these are smaller than the point size.

**Table 3:** Self-Diffusion Coefficients *D*_[C2mim][OAc]_ for the Pretreated Wool Yarn at Different Reference Temperatures

reference temperature (°C)	*D*_[C2mim][OAc]_ (10^–13^ m^2^ s^–1^)
50	1.06 ± 0.06
60	1.62 ± 0.02
70	3.13 ± 0.04
80	8.1 ± 0.1
90	12.4 ± 0.2
100	26.1 ± 0.4
110	41.5 ± 0.6

### TTS of Pretreated Cashmere Yarn

The cashmere yarn (CY) was treated first by NaHSO_3_ and then partially dissolved in [C2mim]­[OAc] according to the process explained in the Methods section. The samples were then dissolved at a range of temperatures and times to cover the low-temperature (LT) and high-temperature (HT) regimes, which were found in the dissolution system of cashmere yarn/[C2mim]­[OAc] above. Figure 12 shows the analysis process of the TTS by taking the set of the data at 70 °C as a reference temperature and aiming to calculate the dissolution activation energy of the pretreated cashmere yarn (PCY) in [C2mim]­[OAc] and then comparing the result to the cashmere yarn dissolution system. The measured cross-sectional area of PCY samples was smaller, compared to CY at the low-temperature regime (50, 55, and 60 °C) for the same dissolution times, which is hypothesized to be rate limited by the disulfide bonds. However, the change in the size of the dissolved PCY at the temperatures ≥70 °C did not significantly change compared to the dissolved CY for the same dissolution time. Interestingly, 70 °C is the crossover temperature in the Arrhenius graph of the CY dissolution system and the above-high-temperature (HT) regime is the temperatures ≥70 °C, which is believed to be the rate limited by hydrogen bonds (see Figure 12). The HT regime appears to be unaffected by the pretreatment with NaHSO_3_, whereas merino wool is affected. It was suggested that this is because wool has higher mol % of cystine than cashmere, which are 11.2 and 6 mol % respectively.^55^ By using eq 7, the dissolution activation energy of the PCY is calculated as 62 ± 4 kJ/mol, which is closer to that of the high-temperature regime of the CY/[C2mim]­[OAc] dissolution system (see Figure 12). This could mean that the energy barrier of the LT regime is lowered by the reducing agent treatment.

**Figure 12:**
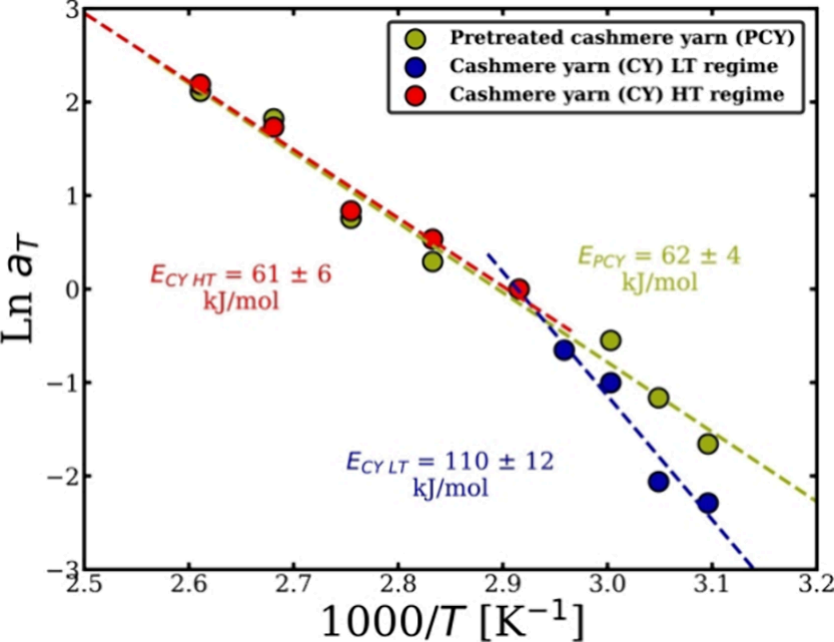
Arrhenius plot of the CY showing the two regimes and of the PCY showing one regime. All the errors were calculated, but, in some cases, these are smaller than the point size.

The relative dissolution rate curve between PCY and the CY shows that the PCY dissolved faster at temperatures below 70 °C and no significant changes at temperatures ≥70 °C (see Figure 13). The speed of the dissolution can be calculated by plotting the linear time master curve of the CY and the PCY at the same reference temperature on one plot. CY data can then be fixed and the PCY can be multiplied by a number to make the data overlap with each other; this number is called the relative dissolution rates.

**Figure 13:**
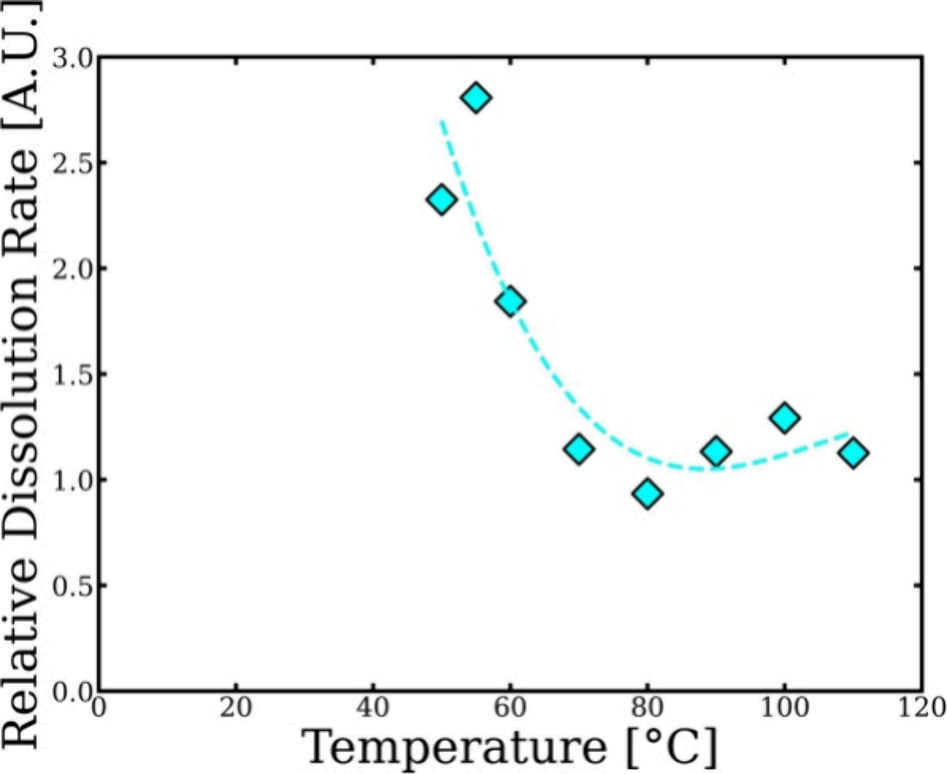
Relative dissolution rate of the PCY in relation to the CY at different temperatures within the system. Polynomial fit used to guide the eye.

The self-diffusion coefficient of [C2mim]­[OAc] in pretreated cashmere yarn was calculated using the same method explained above (see Table 4). It can be seen that the diffusion coefficients for the merino wool system are approximately twice as large as those for the cashmere system. This could be due to differences in the molecular weight of the keratin molecules between the two systems, through which the IL must diffuse. Then, the diffusion activation was calculated using the linear relation of the PCY on the Arrhenius plot (Figure 14b and eq 8) and found to be exactly the same as the dissolution activation energy *E*_D PCY_ = 62 ± 4 kJ/. The data of the Arrhenius plot for the CY and the PCY were plotted together to see that the reducing agent had impacted the dissolution at the temperatures below 70 °C and lowered the activation energy.

**Figure 14:**
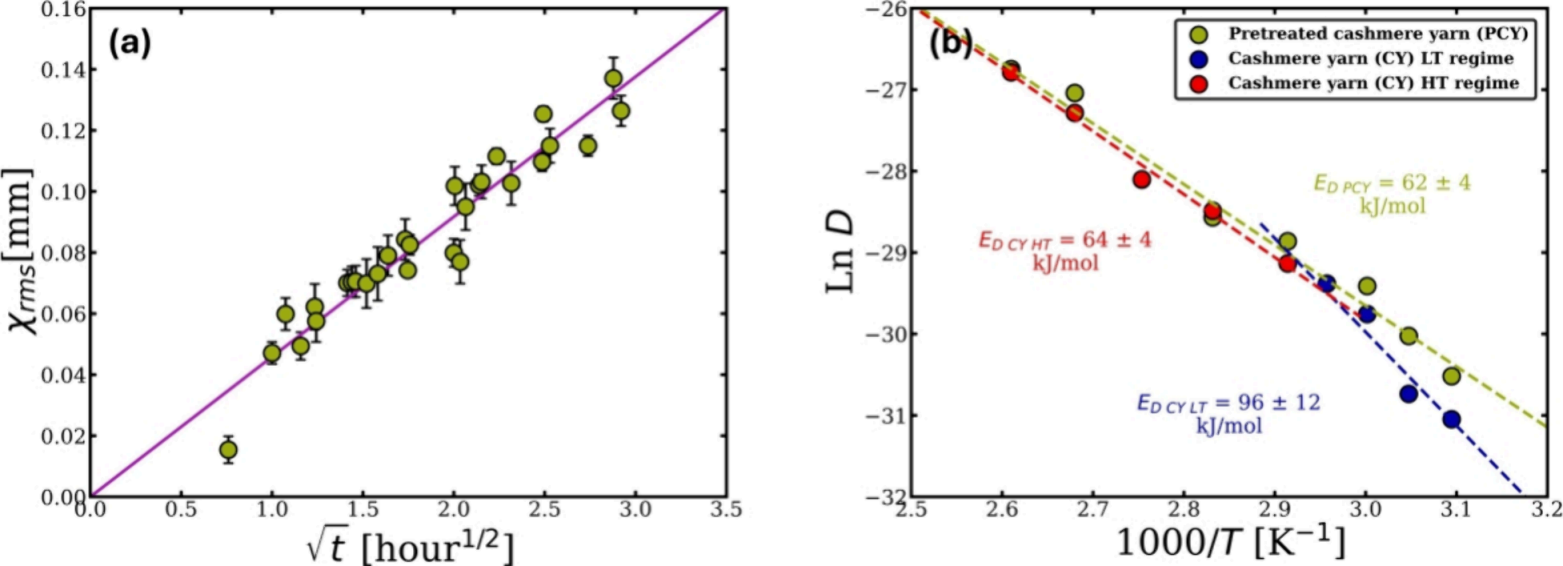
(a) Plot showing the relation between thickness loss of the PCY against $\sqrt{t}$ at 70 °C. (b) Arrhenius graph showing the comparison between the diffusion activation energies of CY and PCY and the value of each energy on the plot. All the errors were calculated, but, in some cases, these are smaller than the point size.

**Table 4:** Self-Diffusion Coefficients for the Pretreated Cashmere Yarn at Different Reference Temperatures

reference temperature (°C)	*D*_[C2mim][OAc]_ (10^–13^ m^2^ s^–1^)
50	0.560 ± 0.001
55	0.91 ± 0.01
60	1.69 ± 0.03
70	2.92 ± 0.05
80	3.92 ± 0.06
90	6.2 ± 0.1
100	18.1 ± 0.3
110	24.2 ± 0.4

## Conclusions

In this article, we report the partial dissolution behavior of cashmere (CY) and merino wool yarns (WY) in the IL 1-ethyl-3-methylimidazolium acetate [C2mim]­[OAc] at different temperatures for a range of dissolution times periods, both with and without sodium bisulfite pretreatment of the yarns. According to the yarn optical images in Figure 2, the cross-sectional areas of yarns were found to decrease in size during the dissolution/coagulation process, so it was used as a parameter to track the dissolution. By varying the dissolution temperature, the time scales for the decrease in yarns size was significantly affected, with higher temperatures accelerating the rate of reduction, as illustrated in Figure 3a. This observation suggested time–temperature equivalence, which was later confirmed through data shifting in the natural logarithmic time domain. Once shifted, the temperature-dependent data sets aligned, forming a single master curve. The shift factors used to create the master curve were plotted against the inverse of their corresponding temperatures.

This revealed that the rate of dissolution of CY can be interpreted to have two distinct regimes; we proposed that one limiting factor was disulfide bonds at low temperatures, and the second limiting factor was hydrogen bonds at high temperatures, with a transition temperature at 70 °C. The two activation energies are *E*_CY LT_ = 110 ± 12 kJ/mol and *E*_CY HT_ = 61 ± 6 kJ/mol, which follow the results in our previous study of the dissolution behavior of WY in [C2mim]­[OAc], which had different activation energies values. To test this, we used sodium bisulfite as a pretreated agent to cleave the disulfide bonds of CY and WY and investigated how this would impact the rate of dissolution. The FTIR spectrum for the pretreated samples exhibited distinctive transmitted intensity that appeared around 1023 cm^–1^, which was a sign of the reduction of the disulfide bonds.

Using the same dissolution experiment method and time–temperature superposition analysis, the temperature dependence of the dissolution rate of the pretreated cashmere yarn (PCY) and the pretreated merino wool (PWY) in [C2mim]­[OAc] were found to have one regime with a single dissolution activation energy. Interestingly, the dissolution activation energies of the PWY and PCY are quite similar, being *E*_PWY_ = 66 ± 3 kJ/mol and *E*_PCY_ = 62 ± 4 kJ/mol, respectively; these energies are approximately half of the activation energies value of the low-temperature regime of the nontreated yarn samples. This strongly supports our hypothesis that the pretreatment removed or reduced the effect of the disulfide bonds, resulting in only one activation energy being obtained.

A further advanced analysis was conducted using the produced master curves to calculate the self-diffusion coefficient of [C2mim]­[OAc]. This was done by modeling the thickness loss of the partially dissolved yarn in [C2mim]­[OAc], and the system was found to be a diffusion-limited process. This also enabled us to calculate the diffusion activation energy of the different types of yarn/[C2mim]­[OAc] system studied here. This research provides important insights into understanding the dissolution of different types of animal hair, which could lead to improvement in the recycling of textile waste materials through the production of biopolymer composites.

## Data Availability

The data associated with this paper are openly available from the University of Leeds Data Repository, https://doi.org/10.5518/1620
